# Development of genodynamic metrics for exploring the biophysics of DNA polymorphisms

**Published:** 2014-11

**Authors:** James Lindesay, Tshela E Mason, William Hercules, Georgia M Dunston

**Affiliations:** 1 Computational Physics Laboratory, Department of Physics and Astronomy, Howard University, Washington, DC, 20059, U.S; 2 National Human Genome Center, Howard University, Washington, DC, 20060, U.S; 3 Department of Microbiology, Howard University, Washingto on, DC, 20059, U.S

**Keywords:** Information theory, entropy, genomic variation, biological information, genodynamics

## Abstract

Single nucleotide polymorphisms (SNPs) represent an important type of dynamic sites within the human genome. These common variants often locally correlate within more complex multi-SNP haploblocks that are maintained throughout generations in a stable population. Information encoded in the structure of SNPs and SNP haploblock variation can be characterized through a normalized information content metric. Genodynamics is being developed as the analogous “thermodynamics” characterizing the state variables for genomic populations that are stable under stochastic environmental stresses. Since living systems have not been found to develop in the absence of environmental influences, this paper describes the analogous genomic free energy metrics in a given environment. SNP haploblocks were constructed by Haploview v4.2 for five chromosomes from phase III HapMap data, and the genomic state variables for each chromosome were calculated. An *in silico* analysis was performed on SNP haploblocks with the lowest genomic energy measures. Highly favorable genomic energy measures were found to correlate with highly conserved SNP haploblocks. Moreover, the most conserved haploblocks were associated with an evolutionarily conserved regulatory element and domain.

## INTRODUCTION

The human genome consists of 3 billion nucleotides, most of which are fixed alleles. A significant number of sites (about 0.1%) consist of single nucleotide polymerphisms (SNPs) non-randomly distributed across the human genome. SNPs are (usually) bi-allelic dynamic sites on the human genome whose allelic distribution reflects the homeostasis of a population within a given environment (Dunston et al., 2014). Here, environment will refer not only to geographic or geophysical parameters, but also to the complete interface of the population to biologic and evolutionary stresses. The defining characteristics of a population are directly reflected within genomic information measures that are maintained throughout generations. Populations are here defined by the maintained order and diversity of the whole genome in its environment.

In developing metrics for the interaction of the human genome with its environment, the genomic environment is the stochastic bath driving variations within a locally viable population. SNPs are dynamic sites that are often highly correlated into SNP haplotypes maintained with fixed frequencies within a given stable population. Combinations of SNPs that are very highly correlated within a population are said to be in linkage disequilibrium (LD). It should be noted that certain SNP allelic combinations never appear within the population. Therefore only certain SNP haplotypes are biologically viable and generationally maintained.

The dynamically independent statistical micro-states are SNP haplotypes together with SNP sites that are not in LD with any other SNPs. The linkage of several SNPs as conserved units that are passed between generations represents a type of statistical phase transition in forming complex dynamic units for a population within a given environment. It is therefore very useful to develop information metrics for SNP microstates that can quantify viable sequence variation in the human genome.

## What is genodynamics?

Genodynamics explores nucleotide structure-function relationships of common sequence variation and population genetics, grounded in first principles of thermodynamics and statistical physics (Lindesay et al., 2012). Our use of the term “genodynamics” is conceptually unrelated to and derived totally independent of any prior use of this term in the published literature. Using genodynamics, we study the informatics of SNPs as dynamic sites in the genome. Viewing structural configurations of SNPs as complex dynamical systems, we earlier developed and utilized the normalized information content (NIC) as a biophysical metric for interrogating the information content (IC) present in SNP haploblocks. SNP haploblocks are defined by the location of the distribution of SNP haplotypes in the genome. The NIC metric, derived from Boltzman’s canonical ensemble and used in information theory, facilitates translation of biochemical DNA sequence variation into a biophysical metric for examining ‘genome-environment interactions’ at the nucleotide level. From this biophysical vantage point, the genome is perceived as a dynamic information system defined by patterns of SNP and SNP haploblock variation that correlate with genomic energy units (GEUs), herein introduced and developed. The quantification of structural configurations encoded in SNP microstates using GEUs provides an additional biophysical metric for interrogating and translating the biology of common sequence variation.

## MATERIALS AND METHODS

### Entropy and information

Information can be quantified in terms of the maintained order of a given system. In the physical sciences, the concept of *entropy* quantifies the dis-order of a physical system (Susskind and Lindesay, 2005). Therefore, entropy can serve as an additive measure of genodynamic variation within a population. This is done by taking the logarithm of multiplicative independent probabilities *p_h_*, which define the *surprisals* log_2_ *p_h_*. The specific (or per capita) entropy of a SNP haploblock consisting of a set of strongly depen-dent bi-allelic SNPs is taken to be the statistical average of this additive measure:

(1) $$s^{\left(H\right)}\equiv -\sum_{h}^{2^{n^{\left(H\right)}}}p_{h}^{\left(H\right)}\;\log_{2}p_{h}^{\left(H\right)}$$

where *n^(H)^* is the number of bi-allelic SNP locations in haploblock H, and $p_{h}^{\left(H\right)}$ represents the probability (frequency) that haplotype *h* occurs in the population. This measure of maintained (dis)order takes the value of zero for a completely homogeneous population with only one haplotype (since for $p_{h}^{\left(H\right)}=1,\;\log_{2}\;p_{h}^{\left(H\right)}=0$), while it takes the value $s_{\max}^{\left(H\right)}=n^{\left(H\right)}$ for a completely stochastic distribution of all SNP alleles with all mathematically possible SNP haploblocks occurring with equal likelihood

$$p_{h}^{\left(H\right)}={\left(\frac{1}{2}\right)}^{n^{\left(H\right)}}$$

For bi-allelic SNPs that are not in LD, there are only 2 possible states at that location. Therefore, the specific entropy of the SNP location *(S)* takes the form:

(2) $$s^{\left(S\right)}\equiv -\sum_{a=1}^{2}p_{a}^{\left(S\right)}\;\log_{2}p_{a}^{\left(S\right)}$$

where $p_{a}^{\left(S\right)}$ represents the probability (frequency) that allele *a* occurs in the population. As defined here, the entropy has no dimensional units. The total specific entropy of the genome in the specified environment is given by the sum over all genetically viable blocks, including correlated SNPs in the haploblocks, along with individual SNPs between the haploblocks that are not in LD,

(3) $$s_{\mathrm{Genome}}=\sum_{H}s^{\left(H\right)}+\sum_{S}s^{\left(S\right)}$$

This insures that all dynamic SNP degrees of freedom are included in calculating the genomic entropy. Because this entropy measure is additive, it also quantifies the entropy within any region of the genome. The overall entropy of a population distribution is proportional to the size of the population *N_Population_*, that is, *S_Genome_* = *N_population_ S_Genome_*, making entropy an extensive state variable.

Since entropy is a measure of the disorder of a distribution, a system with maximum disorder is one of maximum entropy. In contrast, the information content of a distribution is measured by the degree of order that the distribution has relative to a completely disordered one, that is, the difference between the entropy of the distribution and that of a completely disordered distribution; *IC* = *S*_max_ − *S*. Such an information measure is likewise additive due to the additive nature of the entropy (Lindesay, 2013).

In our previous work (Lindesay et al., 2012), a normalized information metric was developed as a means of comparison of the information contained within specific regions of the genome, as well as between various populations. This NIC value ranges between 0 and 1, where a value of zero indicates a completely random allelic distribution, while a value of unity represents a homogeneous allelic distribution without variation. The NIC for a given SNP haploblock (H) is defined by:

(4) $${\mathrm{NIC}}^{\left(H\right)}\equiv \frac{s_{\max}^{\left(H\right)}-s^{\left(H\right)}}{s_{\max}^{\left(H\right)}}=\frac{n^{\left(H\right)}-s^{\left(H\right)}}{n^{\left(H\right)}}$$

One should note that unlike the information content, NIC is not an additive measure for multi-SNP haploblocks. The information measure for the whole genome in an environment must be calculated using the total number of SNP locations in the genome, as well as the total specific entropy of the genome.

### Statistical energetics

The statistical “genomic energy” of a population in a given environment is expected to be an additive (extensive) state variable that depends upon the entropy, the populations of various allelic constituencies, and possibly the “genomic volume” of the environment, if population pressures have a significant effect on the environment. The functional dependence of the contribution of haploblock H to the average genomic energy *U* can be expressed using the differential expression:

(5) $${dU}^{\left(H\right)}\equiv T_{E}\;{dS}^{\left(H\right)}+\sum_{h}\mu_{h}^{\left(H\right)}{dN}_{h}^{\left(H\right)}-\Pi_{E}^{\left(H\right)}{dV}^{\left(H\right)}$$

where *T_E_* represents an environmental potential (which is conjugate to the entropy state variable), $\mu_{h}^{\left(H\right)}$ represents the haplotype potential of haplotype *h* in SNP haploblock H, $N_{h}^{\left(H\right)}=p_{h}^{\left(H\right)}\;N_{\mathrm{Population}}$ represents the population of haplotype *h*, and $\Pi_{E}^{\left(H\right)}$ represents any “pressure” by the haploblock on the environment that would result in expansion of the genomic “volume” *V^(H)^*. In all subsequent expressions, any genomic effects that would modify the genomic volume will be neglected $\Pi_{E}^{\left(H\right)}{dV}^{\left(H\right)}=0$.

As is the case for thermodynamics and statistical physics, it is quite convenient to define an additive free energy state variable that is most naturally expressed as a function of the potential of the environmental bath *T_E_* and the populations, through the Legendre transformation

(6) $$F^{\left(H\right)}\equiv U^{\left(H\right)}-T_{E}S^{\left(H\right)}\;\;\;,\;\;\;dF^{\left(H\right)}\equiv -S^{\left(H\right)}{dT}_{E}+\sum_{h}\mu_{h}^{\left(H\right)}{dN}_{h}^{\left(H\right)}$$

A focus on the free energy as the fundamental dynamic state variable has the advantage of inherently including environmental-genomic interchanges as necessary considerations in describing the dynamics. It is a particularly convenient parameter for describing dynamics in a fixed environmental bath for which *dT_E_ = 0*. As one recognizes that living cells have evolved their cellular functions within the warm, wet physiologic environment, one can safely conclude that a homeostatic living population distribution has evolved directly in association with the ecosystem within which it is being characterized. Thus, we assert that the evolution of living populations cannot be separated from their interchanges with the environment. In a statistical environment that is stochastically varying, it is the genomic free energy rather than the genomic energy that is minimized. The genomic free energy is a state variable that balances between conservation and variation of SNP haplotypes within an environment. Minimizing the genomic free energy optimizes the population’s survivability under environmental stresses, establishing the balance between conservation and variation in the dynamics of the population distribution.

For the genome, only the site locations and bi-allelic nature of the specific SNPs are conserved parameters. In addition, phase transitions involving the stability of SNP haploblock structures are common between differing populations_,_ resulting in non-conservation of the number and SNP composition of the haploblocks. This is in marked contrast with the standard micro-units in statistical physics, whose universal energy states are only weakly dependent upon the environment, and have well defined conservation properties with regards to the creation of new states (or changing dynamic degrees of freedom). Therefore, rather than seeking universal energy measures that are independent of the genomic environment, the emphasis here will be based on establishing convenient genomic measures of the dynamics that are inseparably coupled with environmental parameters. Since the allelic potentials, given by $\mu_{h}^{\left(H\right)}={\left(\frac{\partial F^{\left(H\right)}}{\partial N_{h}^{\left(H\right)}}\right)}_{T_{E}}$, are the parameters in the environmental bath that dynamically couple to the SNP haplotype unit *h*, the formulation will be developed in a manner that most directly interprets these genomic energy measures.

Using the differential form for the haploblock free energy $dF^{\left(H\right)}=-S^{\left(H\right)}{dT}_{E}+\sum_{h}\mu_{h}^{\left(H\right)}{dN}_{h}^{\left(H\right)}$ from (Equation 6), we can use the expression of the population with haplotype *h* given by $N_{h}^{\left(H\right)}=p_{h}^{\left(H\right)}\;N_{\mathrm{Population}}$ to expand the differential ${dN}_{h}^{\left(H\right)}={dp}_{h}^{\left(H\right)}\;N_{\mathrm{Population}}+p_{h}^{\left(H\right)}{dN}_{\mathrm{Population}}$. Re-writing the variation of the haploblock free energy in terms of the population gives:

(7) $$dF^{\left(H\right)}=\left(-s^{\left(H\right)}{dT}_{E}+\sum_{h}\mu_{h}^{\left(H\right)}{dp}_{h}^{\left(H\right)}\right)N_{\mathrm{Population}}+\left(\sum_{h}\mu_{h}^{\left(H\right)}p_{h}^{\left(H\right)}\right){dN}_{\mathrm{Population}}$$

### Population stability

Values for all of these additive genomic state variables can be likewise assigned to those SNPs that are not in linkage disequilibrium by simply replacing the particular haploblock index *(H)* in any of the previous formulas with the SNP location *(S)*. The total genomic free energy will be a sum over all SNP haploblocks and non-linked SNPs given by:

(8) $$F_{\mathrm{Genome}}=\sum_{H}F^{\left(H\right)}+\sum_{S}F^{\left(S\right)}$$

We further examined the condition that a stable population is defined by the genomic data. Our condition will require that the genomic free energy be a minimum under changes in the population within the local environment when the population is stable, that is, $(\frac{\partial F_{\mathrm{Genome}}}{\partial N_{\mathrm{Population}}})=0$.

The average allelic potential within a SNP haploblock $\sum_{h}\mu_{h}^{\left(H\right)}p_{h}^{\left(H\right)}=\langle \mu^{\left(H\right)}\rangle$ will be referred to as the block potential for haploblock *(H)*, while the average allelic potential at a non-linked SNP location $\sum_{a}\mu_{a}^{\left(S\right)}p_{a}^{\left(S\right)}=\langle \mu^{\left(S\right)}\rangle$ will be referred to as the SNP potential for location *(S)*.

From Equation 7 for the genomic free energy in terms of block potentials and SNP potentials holding the environmental potential and frequencies fixed, the population is seen to be stable if the overall genomic free energy satisfies:

(9) $${\left(\frac{\partial F_{\mathrm{Genome}}}{\partial N_{\mathrm{Population}}}\right)}_{T_{E},p_{h}}=0=\sum_{H}\left(\sum_{h}\mu_{h}^{\left(H\right)}p_{h}^{\left(H\right)}\right)+\sum_{S}\left(\sum_{a}\mu_{a}^{\left(S\right)}p_{a}^{\left(S\right)}\right)=\sum_{H}\langle \mu^{\left(H\right)}\rangle +\sum_{S}\langle \mu^{\left(S\right)}\rangle \equiv \mu_{\mathrm{Genome}}$$

where *a* shows the particular allele at SNP location (S).

Our population stability condition incorporates Hardy-Weinberg equilibrium (Hardy, 1908; Weinberg, 1908) in population genetics. Hardy-Weinberg equilibrium asserts that in order for the genomic distributions to meaningfully represent a stable population, the various frequencies of haplotypes and alleles should be stable. Since the frequencies directly determine the block and SNP potentials, a requirement that these environmentally dependent potentials remain fixed and sum to zero satisfies Hardy-Weinberg equilibrium. Such stable populations maintain the distribution of SNPs throughout the generations within the given environment. The genomic average allelic potential *μ_Genome_*, which is the sum over all block potentials and SNP potentials, is seen to vanish if the population does not increase or decrease. This means that a stable population is balanced with regards to its overall sum over allelic potentials, *μ_Genome_*=0. The genomic free energy is lowered by a population with negative overall genomic potential *μ_Genome_*<0 if its size increases, while if *μ_Genome_*>0 the genomic free energy is lowered if the population decreases.

As is the case of thermodynamics, the additive allelic potentials $\mu_{h}^{\left(H\right)}$ are expected to scale relative to the environmental parameter *T_E_*, and allelic potential differences should directly reflect in the ratio of the frequencies of occurrence of those haplotypes within the population. A functional form that has these properties is given by:

(10) $$\frac{\mu_{h2}^{\left(H\right)}-\mu_{h1}^{\left(H\right)}}{T_{E}}=-\log_{2}\frac{p_{h2}^{\left(H\right)}}{p_{h1}^{\left(H\right)}}$$

The genomic energy labeled μ̃ will be defined as the unique allelic potential that will insure that a single (bi-allelic) SNP will be in its state of highest variation $\tilde{p}=\frac{1}{2}$ within the given species. Similarly, a haploblock with *n^(H)^* SNPs in its state of highest variation with all mathematically possible haplotypes occurring with frequencies $p_{h}^{\left(H\right)}={\left(\frac{1}{2}\right)}^{n^{\left(H\right)}}$ will have a block potential of *n*(*H*) *μ̃*. The unit *μ̃* will be universal across all populations of a given species, but likely differs between species. Solving the previous equation, the allelic potential of the haplotype *h* or allele *a* in an environmental bath characterized by environmental potential *T_E_* can be expressed as:

(11) $$\begin{array}{l} \mu_{h}^{\left(H\right)}=(\tilde{\mu }-T_{E})n^{\left(H\right)}-T_{E}\log_{2}p_{h}^{\left(H\right)}\\ \mu_{a}^{\left(S\right)}=(\tilde{\mu }-T_{E})-T_{E}\log_{2}p_{a}^{\left(S\right)} \end{array}$$

where the allelic potential for a single non-linked SNP location *(S)* has *n*^(^*^S^*^)^ = 1. Using our identifications, a lower allelic potential is then associated with a higher conservation of the SNP haplotype within the population, as high entropy is associated with large variation within the population. The ability to assign a well defined genomic energy measure for an individual haplotype once the environmental potential *T_E_* is known allows this formulation to establish biophysical measures beyond statistical statements about the population as a whole.

Haplotypes and alleles with high genomic energy are highly unfavorable in the given environment. The value of the allelic potential $\mu_{a}^{\left(S\right)}$ that fixes a single non-linked SNP location *(S)* into a given allele ( $p_{a}^{\left(S\right)}\to 1$) will be defined to be the fixing potential in the given environment. If the allele has this potential, it is homogeneous throughout the population. This value is directly related to the environmental potential through:

(12) $$\mu_{\mathrm{Fixing}}=\tilde{\mu }-T_{E}$$

Thus, the allelic potential of any single SNP location cannot be determined to be less than the fixing potential through measurements in a single environment.

The population stability condition $\mu_{\mathrm{Genome}}=\sum_{H}\langle \mu^{\left(H\right)}\rangle +\sum_{S}\langle \mu^{\left(S\right)}\rangle =0$ can be used to determine the environmental potential. By substituting the forms of the allelic potentials $\mu_{h}^{\left(H\right)}$ and $\mu_{a}^{\left(S\right)}$ expressed in terms of the probabilities into the population stability condition, an explicit expression of the environmental potential can be obtained:

(13) $$T_{E}=\frac{\tilde{\mu }n_{\mathrm{SNPs}}}{n_{\mathrm{SNPs}}-s_{\mathrm{Genome}}}=\frac{\tilde{\mu }}{{\mathrm{NIC}}_{\mathrm{Genome}}}$$

where $n_{\mathrm{SNPs}}\equiv \sum_{H}n^{\left(H\right)}+\sum_{S}n^{\left(S\right)}$ is the total number of SNP locations on the genome. The average allelic potential for a given SNP haploblock, which has been defined as the block potential of that haploblock, then satisfies:

(14) $$\langle \mu^{\left(H\right)}\rangle =\left(1-\frac{{\mathrm{NIC}}^{\left(H\right)}}{{\mathrm{NIC}}_{\mathrm{Genome}}}\right)n^{\left(H\right)}\tilde{\mu }$$

which has been obtained by simply taking the statistical average of the allelic potentials in (Equation 11), and substituting the expression for the environmental potential in terms of the genomic normalized information content.

These measures of genomic potentials have several convenient features:

1. The environmental potential *T_E_* is inversely proportional to the IC of the whole genome. Low IC results from a high environmental potential, while a completely conserved genome has the lowest possible environmental potential, which we can define to have the value of one genomic energy unit *μ̃* =1 GEU. A population with a completely disordered genomic distribution would inhabit an environment with infinite environmental potential.
2. SNP haploblocks that are highly conserved relative to the whole genome will have negative block potentials, while those that are highly varying will have positive block potentials. The block potentials typically lie within the range specified by *n*^(^*^H^*^)^ *μ_Fixing_* ≤ 〈*μ*^(^*^H^*^)^〉 ≤ *n*^(^*^H^*^)^ *μ̃* (although the lower bound is not rigorously required).
3. The number of highly correlated SNPs within the haploblock *n*^(^*^H^*^)^ amplifies SNP haploblock allelic potentials.

One should note that while the environmental potential *T_E_*, the block potentials 〈*μ*^(^*^H^*^)^〉 and the SNP potentials 〈*μ*^(^*^S^*^)^〉 can only be defined for a population, the individual allelic potentials $\mu_{h}^{\left(H\right)}$ and $\mu_{a}^{\left(S\right)}$ define an overall allelic potential for each individual in the population:

(15) $$\mu_{\mathrm{indidual}}=\sum_{H}\mu_{h}^{\left(H\right)}+\sum_{S}\mu_{a}^{\left(S\right)}$$

where the SNP haplotypes *h* and alleles *a* are unique to the individual. An individual’s overall allelic potential is not a universal parameter, but rather depends strongly upon the environment. Thus, the overall allelic potential of an individual is not an essentially fixed microphysical genomic energy state, in contrast to the energetics of particles in statistical physics. An environment within which an individual haplotype or allele has a negative allelic potential tends to conserve that characteristic, while a haplotype or allele that has a positive allelic potential provides diversity and viable genomic variation within that environment. The value of the allelic potential gives a direct measure of the dynamic (un)favorability of a haplotype as a function of the environment.

### Analysis of the block potentials associated with five chromosomes in the human genome

To demonstrate the usefulness of the previously defined genomic state variables, the parameters will be calculated using genomic data for stable populations. We choose to utilize genotype data provided by the HapMap Project on the Yoruba in Ibadan, Nigeria (YRI) and the Utah residents with ancestry from Northern and Western Europe (CEU). Because of the time involved in the calculations, we have chosen representative large, medium and small chromosomes (1, 6, 11, 19, and 22) within the genome to examine the uniformity of the genomic potentials, and comparisons between populations.

Our formulation requires that the SNP haploblock structure that codifies the LD between local SNPs be established for a given population. For this purpose, we used Haploview, which is a software package in the public domain that is in general use. SNP haploblocks were constructed for the representative chromosomes using the confidence interval algorithm developed by Gabriel et al. (2002) in Haploview v 4.2 from HapMap phase III data. Haploview uses a two marker expectation-maximization algorithm with a partition-ligation approach that creates highly accurate population frequency estimates of the phased haplotypes based on the maximum-likelihood as determined from the unphased input (Barrett et al., 2005). Once the block structure of the population has been constructed, we have developed software that takes that data and calculates the genomic state variables for each of the chromosomes. This data was then graphed for analysis. In order to demonstrate the usefulness of the genomic state variables, rather than overwhelm the reader with the abundance of data contained within all the chromosomes that have been examined, the parameters are here demonstrated for chromosome 6 of both the examined populations. Additionally, an *in silico* analysis was performed on SNP haploblocks with the lowest genomic energy measures on chromosome 6 scanning for associated regulatory elements, signatures of positive selection, protein domains, molecular functions and biological processes using publically available bioinformatics tools (Boyle et al., 2013; Brown et al., 2013; Friedman et al., 2014; Gagen et al., 2005 ;Genome Bioinformatics Group of UC Santa Cruz, 2013; Greer et al., 2014; Lee and Shatkay, 2013; Sandelin et al., 2013; Sherry et al., 2012; Sigrist et al., 2013; Thorisson et al., 2012; Wu et al., 2013; Zhang et al., 2013).

It will be assumed that the environmental potential *T_E_* that would be calculated from the NIC of the whole genome does not differ significantly from that calculated using the five chromosomes. This parameter takes the value *T_E_*_,(YRI)_=1.26 GEUs for the YRI population, and *T_E_*_,(CEU)_=1.12 GEUs for the CEU population.

## RESULTS

**Figure 1:**
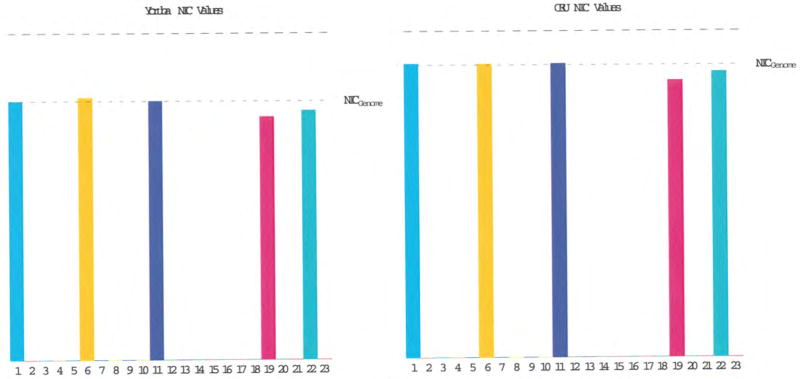
Analysis of NIC values for chromosomes 1, 6, 11, 19 and 22 in the YRI and CEU populations.

The distributions of the NIC values across the genomes of the YRI and CEU populations are demonstrated in Figure 1. The overall distributions of NIC values for these two populations have a similar chromosomal distribution pattern despite the NIC values for the CEU population being higher than those for the YRI. In the CEU population, the NIC values for the chromosomes studied are as follows: NIC_1_≅0.90, NIC_6_≅0.90, NIC_11_≅0.89, NIC_19_≅0.85 and NIC_22_≅0.87; while the NIC values for the YRI population are: NIC_1_≅0.79, NIC_6_≅0.80, NIC_11_≅0.79, NIC_19_≅0.74 and NIC_22_≅0.76.

**Figure 2:**
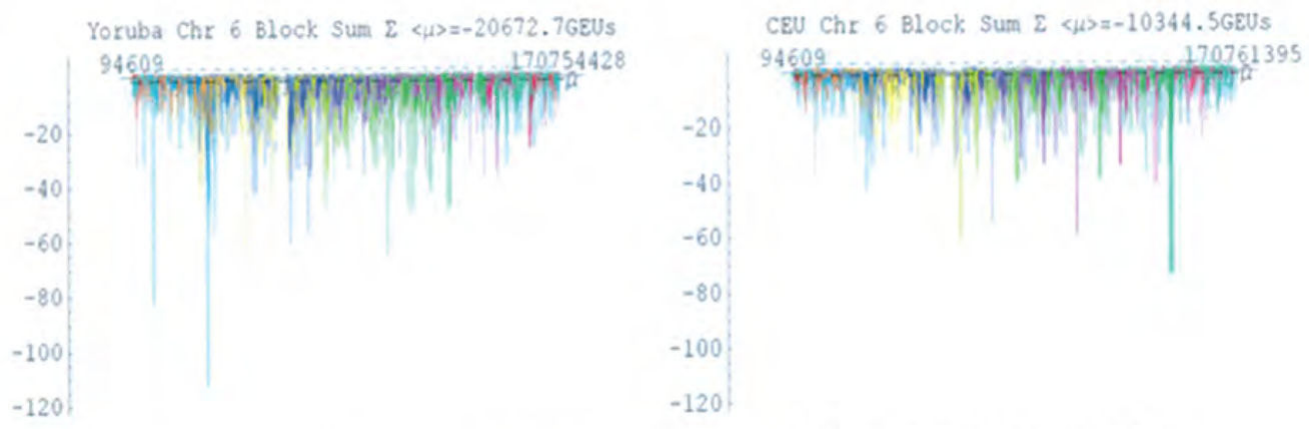
Analysis of the genomic energy measurements for chromosome 6 in the YRI and CEU populations.

The genomic energy spectra for chromosome 6 of the YRI and CEU populations are demonstrated in Figure 2. In the YRI population, there were 6,810 SNP haploblocks with positive potentials and 6,738 with negative potentials. In comparison, the CEU population had 5,160 SNP haploblocks with positive potentials and 3,600 with negative potentials. No highly varying SNP haploblock has a block potential significantly larger than the environmental potential *T_E_*.

**Figure 3:**
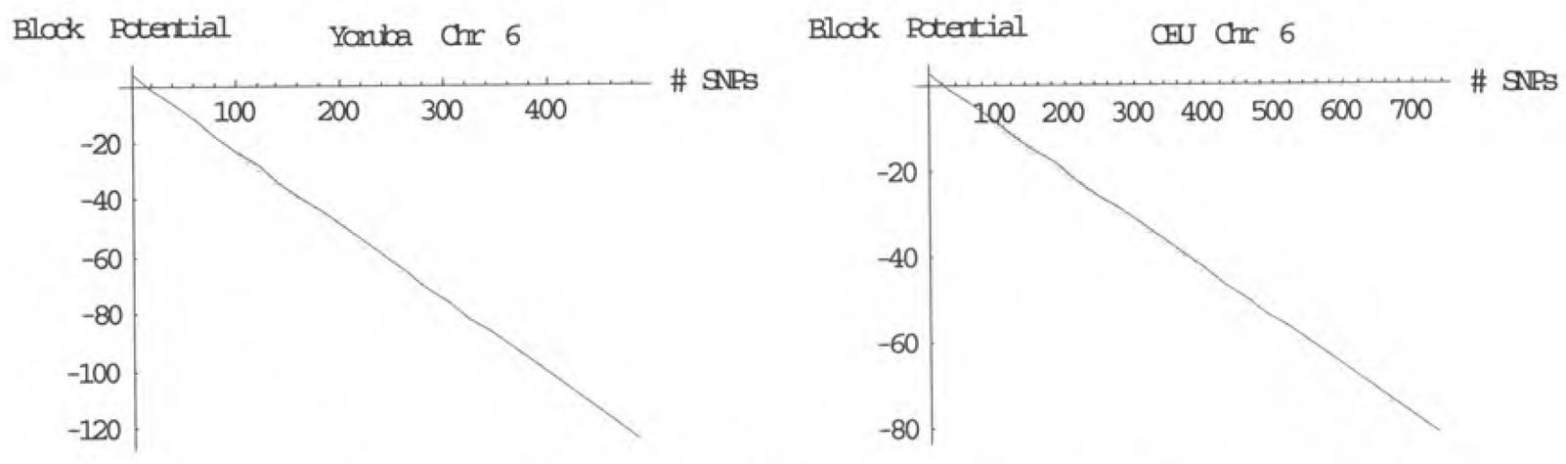
Comparison of the genomic energy spectra for chromosome 6 in the YRI and CEU populations.

The block potential as a function of the number of SNP locations in the haploblock for each population was examined, and this is illustrated in Figure 3. A set of good fits for the block potentials for large haploblocks are given by:

$$\begin{array}{l} \mu_{\mathrm{VRI}}^{\left(H\right)}≅\left(3.84-0.26n^{\left(H\right)}\right)\mathrm{GEUs}\\ \mu_{\mathrm{CEU}}^{\left(H\right)}≅\left(2.81-0.11n^{\left(H\right)}\right)\mathrm{GEUs} \end{array}$$

where again, *n^(H)^* is the number of SNP locations in haploblock H. This indicates that those SNP haploblocks that are highly conserved are seen to have block potentials that approach the fixing potential μ_fixing_ for the given specific environment times the number of SNP locations in the block.

**Figure 4:**
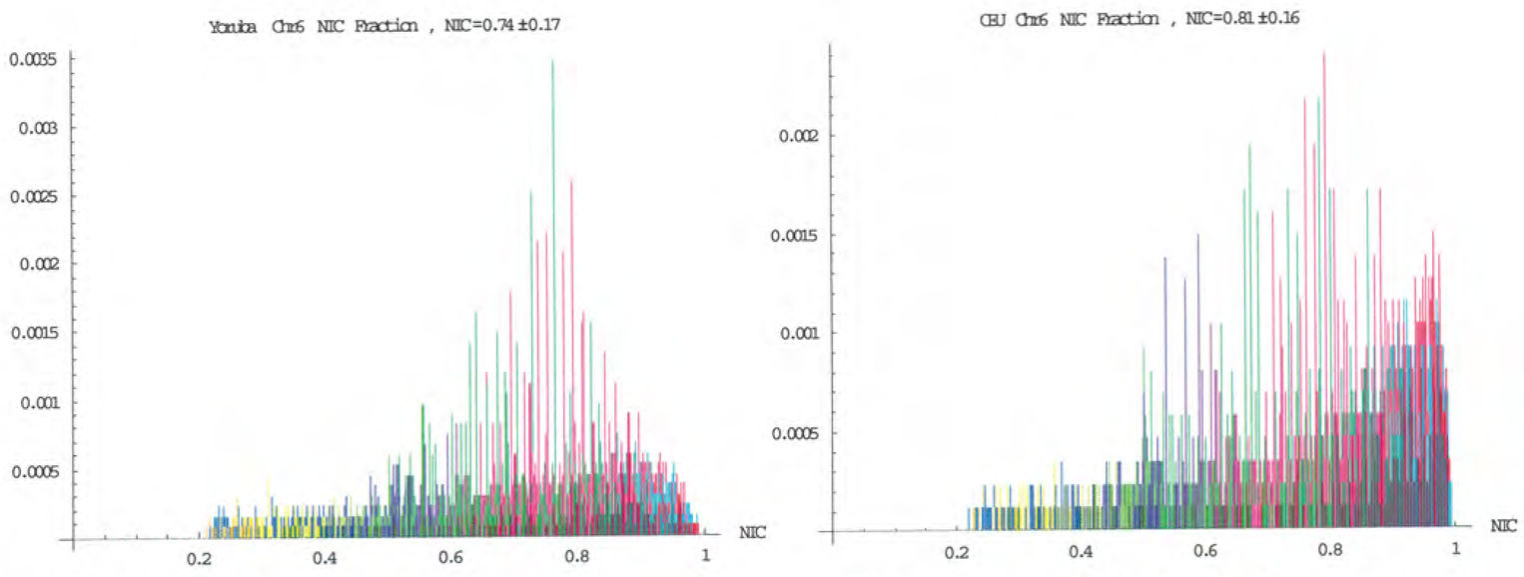
Comparison of the informatics of chromosome 6 in the YRI and CEU populations. The NIC value for the YRI population is 0.74±0.17 and 0.81±16 for the CEU population.

The number of SNP haploblocks within given intervals of NIC as a measure of the proportion of haploblocks that maintain a specific degree of variation were plotted, and bar graphs of these proportions are demonstrated in Figure 4. The distribution for the CEU population is shifted relative to that of the YRI population, so that there are an increased number of SNP haploblocks with higher NIC values. Also, it was noticed that there are no SNP haploblocks with a NIC value lower than ~0.2.

The block spectrum of SNP haploblocks in the MHC region for the populations of interest was examined. The genomic energies for the YRI population are $\sum_{H}\mu^{\left(H\right)}≅-2293\mathrm{GEUs}$ as compared to CEU whose genomic energies are $\sum_{H}\mu^{\left(H\right)}≅-751\mathrm{GEUs}$. The most highly conserved haploblock found within any of the chromosomes thus far examined is Block 3013 on chromosome 6 in this region of the YRI population. It is worth noting that the most highly conserved haploblock in the CEU population, Block 7016, is also located on chromosome 6 however it is not in the MHC region.

### *In silico* analysis of blocks 3013 in the YRI population and 7016 in the CEU population

Block 3013 is located between 6p22 and 6p21.3 bands (29,960,986-30,043,628) on chromosome 6 (Figure 6a). It has 441 SNP locations, with 226 of them being dynamic. It contributed a highly favorable averaged block potential of −112 GEUs to the overall genomic energy and had a NIC value of 0.991. Block 3013 has 253 SNPs in genes and 188 SNPs in non-genic regions. This block included six genes: (1) Zinc ribbon domain 1 (ZNRD1); (2) ZNRD1-antisense RNA1 (ZNRD1-AS1); (3) Human leukocyte antigen (HLA) complex group 8 (HCG8); (4) Protein phosphatase 1 regulatory inhibitor subunit 11 (PPP1R11); (5) HLA-J and (6) Ring finger protein 39 (RNF39) as shown in Figure 6a.

ZNRD1, PPP1R11 and RNF39 are genes with functional proteins, whereas HCG8 and ZNRD1-AS1 are both non-coding RNAs (ncRNAs). HLA-J is a transcribed pseudogene. ZNRD1, PPP1R11, and RNF39 are highly conserved across species ranging from chimpanzee to zebrafish. These genes also display signatures of positive selection, but only in populations of European descent. Several putative and confirmed transcription factor binding sites (TFBS) are in Block 3013. Also, several broadly conserved microRNAs (miRNAs) are in Block 3013. It is worth noting that the ncRNA, ZNRD1-AS1, is a natural antisense transcript (NAT) that regulates the expression of ZNRD1.

Block 7016 is located on the 6q24 band (145,851,676-146,351,676) on chromosome 6 (Figure 6b). This block contributed a highly favorable averaged block potential of −73.85 GEUs to the overall genomic energy and had a NIC value of 0.995. Block 7016 contains 666 SNPs, with 353 of them being dynamic. This block has 399 SNPs in genes and 267 SNPs in non-genic regions. It included four genes: (1) Epilepsy, progressive myoclonus type 2, Laforin disease [laforin] (EPM2A); (2) Uncharacterized protein (RP11-54515.3); (3) SNF2 histone linker PHD RING helicase E3 ubiquitin protein ligase (SHPRH) and (4) F-box protein 30 (FBXO30) as shown in Figure 6b.

EPM2A, SHPRH and FBXO30 are genes with functional proteins whereas RP11-54515.3 is a ncRNA. The protein coding genes located in this block are also highly conserved across species ranging from chimpanzee to *Arabidopsis thaliana*. Even though the protein coding genes in this block are highly conserved across species, there were no signatures of positive selection associated with any of the genes in this block. There are several putative and confirmed TFBS associated with Block 7016. Like Block 3013, Block 7016 also has several broadly conserved miRNAs. The ncRNA, RP11-54515.3, is also a NAT which regulates the expression of SHPRH and FBXO30.

Listed in Table 1 are the protein domains associated with the genes in Blocks 3013 and 7016, while Table 2 outlines the molecular functions associated with these genes. Table 3 depicts the biological processes associated with the genes in these blocks. With regard to their protein domains, both blocks contain genes with evolutionarily conserved domains which are in lowercase in Table 1. Also, in boldface in Table 1 are the protein domains that both blocks have in common. In Table 2, the molecular functions that are associated with one or more evolutionarily conserved protein domains are in lowercase while those molecular functions that both blocks have in common are in boldface. With regard to their biological processes, there were no commonalities between the two blocks. However, those processes associated with one or more evolutionarily conserved protein domains found in Blocks 3013 and 7016 are in lowercase. It is worth noting that the ncRNAs and pseudogene were excluded from this analysis due to the fact that they are non-coding genes and would lack said domains, functions and processes.

## DISCUSSION

We have developed genomic energy measures for the human genome that relate the distribution of alleles within a stable population to state variables associated with the environment within which that population resides. The state variables defined by common variations utilize the entropy of the statistical distribution of alleles to establish normalized information measures for persistent dynamic units within arbitrary regions of the genome, as well as for the genome as a whole. For our initial analysis, YRI and CEU were chosen as representative populations in or very near homeostasis with their respective environments. Moreover, these populations have significant differences in the degree of variation in SNP allele and haplotype frequencies. As demonstrated in Figure 1, the YRI population has overall greater variation, while the CEU population exhibits more conservation, as quantified by its higher overall NIC. In both populations, it is clear that each of the five chromosomes examined in this study has a NIC value within 10% of the composite NIC value for that population. Also, the larger chromosomes have NIC values that seem to be quite representative of the composite NIC value for that population, while the smaller chromosomes seem to maintain slightly higher variation. Moreover, the relative distribution of conservation amongst the chromosomes seems to take the same shape between the two populations. Whether these features are fundamental properties of the genome remains an unsettled question for further studies. We will first expand our exploration to include all chromosomes for the selected populations; then include all populations we expect to be in environmental homeostasis. The formulation should be applicable to all populations in quasi-homeostasis consistent with publically available genomic distribution data.

We further made comparisons of genomic energy measures between the individual SNP haploblocks within chromosome 6 which is illustrated in Figure 2. We developed a genomic energy spectrum by plotting the block potential of each haploblock in GEUs as a function of its location on the chromosome. Since the block potential is an average of the allelic potentials of the various haplotypes that make up the haploblock, such genomic energy spectra describe the population as a whole. Given that their sum must vanish, it was initially expected that the spectrum would display an even distribution of positive and negative potentials. However, it is clear that although those haploblocks contributing positive block potential are uniformly distributed, those haploblocks contributing negative block potentials are far fewer and more conserved, displaying an inverted Manhattan-plot profile.

There is another interesting characteristic of the block potentials that was seen across all the chromosomes examined. This feature was discovered upon exploring the dependency of the block potentials upon the number of SNP locations within those haploblocks (Figure 3). Although the block potential per SNP varied somewhat for haploblocks containing fewer than ~50 SNPs, the block potential per SNP for larger haploblocks is constant within a given population, regardless of the chromosome examined. The slope of this linear relationship is the fixing potential in the given environment, suggesting that larger haploblocks have been “optimally” shaped by the environment. This is sensible when recognizing that as block size increases, relatively fewer variations remain biologically viable.

It was instructive to directly compare the informatics of chromosome 6 in the two populations. The NIC takes values between zero and one, where a value of zero indicates maximal variation in SNP haplotypes, while a value of one indicates complete sequence homogeneity of the population. We plotted the number of SNP haploblocks within given intervals of NIC as a measure of the proportion of haploblocks that maintain a specific degree of variation (Figure 4). It is clear that the distribution of the NIC values for the CEU population is shifted towards one relative to that of the YRI population. Our prior studies (Lindesay et al., 2012) demonstrated that those SNP haploblocks with low NIC were associated with innate immune regulation and functions that require rapid response to environmental stresses. It is also worth noting that neither population has haploblocks with NIC values lower than ~0.2. This indicates that many mathematically possible variations of alleles within the haploblocks are not viable within these stable human populations.

To further examine the biophysical interpretations of genomic energies, we considered the block spectrum of SNP haploblocks in the MHC region for the populations of interest. A striking feature of comparison between the spectra is that despite the overall higher diversity in the YRI population as quantified in its considerably lower NIC when compared to the CEU population, the YRI population had genomic energies that were considerably more conserved $\sum_{H}\mu^{\left(H\right)}≅-2293\mathrm{GEUs}$ as compared to those of the CE EU population $\sum_{H}\mu^{\left(H\right)}≅-751\mathrm{GEUs}$.

**Figure 5:**
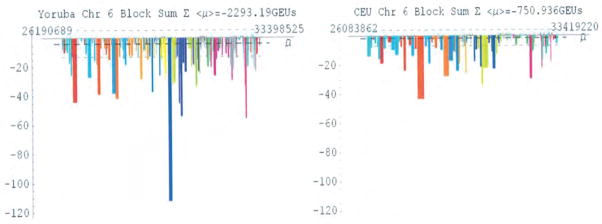
Comparison of the major histocompatibility complex (MHC) region on chromosome 6 for the YRI and CEU populations.

**Figure 6:**
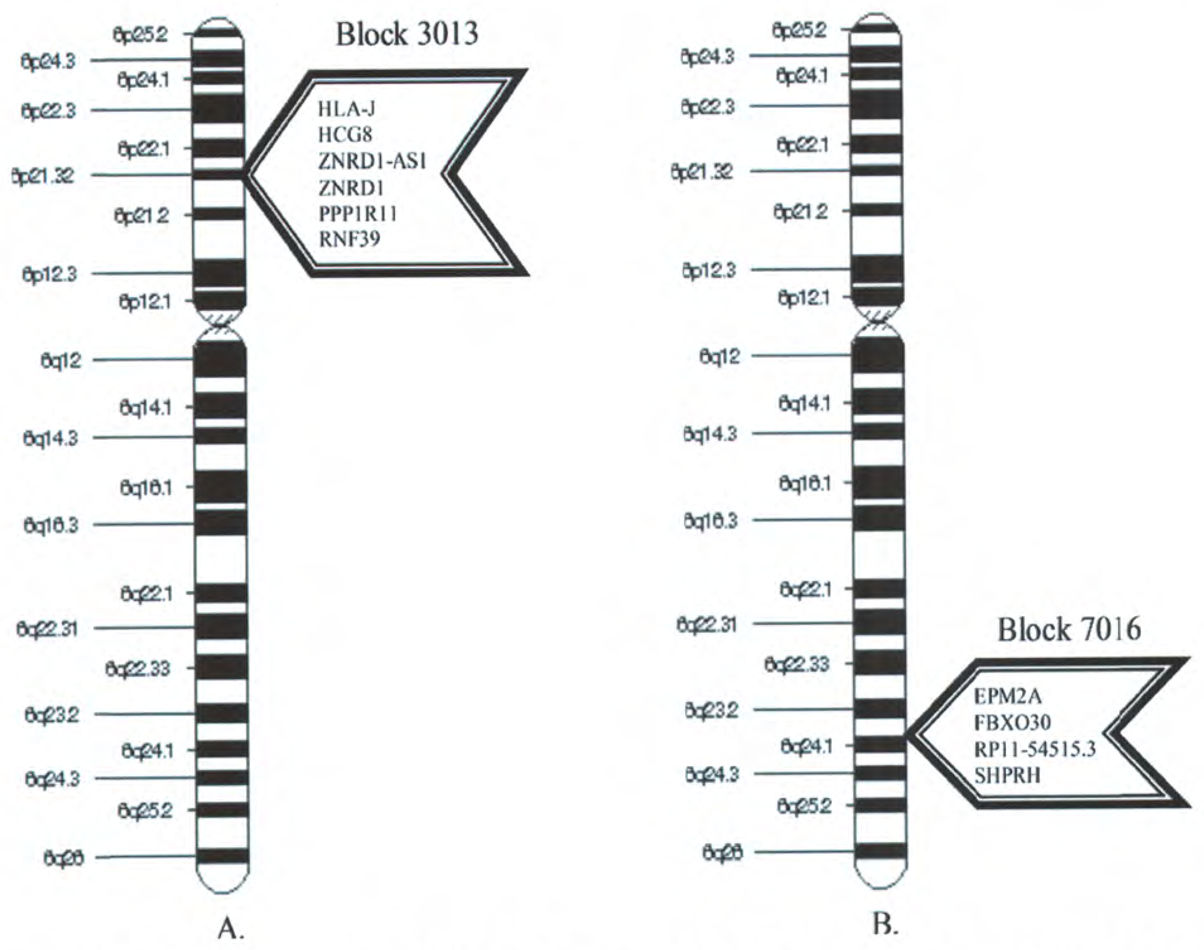
Figures 6a and 6b. The ideogram on the left is of chromosome 6 illustrating the location of Block 3013 in the YRI population, while the ideogram on the right illustrates the location of Block 7016 in the CEU population on chromosome 6.

This is also apparent from the lower value for the average block potential demonstrated by the middle dashed lines in Figure 5. It is intriguing to find that despite the relatively large difference between YRI and CEU in their composite NIC values (~13%), the NIC values of their respective MHC regions were within 2%, indicating comparable normalized information content (NIC). This results in considerably lower GEUs for alleles in the MHC region of the YRI as compared to the CEU population (~300%). Within a given environment, the MHC region seems to adjust its GEUs to conserve its NIC.

Given that this region is important for encoding immune responses, it is expected that it would be particularly sensitive to environmental influences. Surprisingly, the most highly conserved haploblock found within any of the chromosomes thus far examined is the Block 3013 on chromosome 6 in the MHC region of the YRI population, while the most highly conserved haploblock in the CEU population is also on chromosome 6, Block 7016, though this block is not located in the MHC region. Both blocks have NIC values approaching one (complete sequence homogeneity of the population) and the protein coding genes located in these blocks are highly conserved across species, implying that these genes may play a fundamental role in the biological processes necessary for life. The protein coding genes ZNRD1, RNF39 and PPP1R11, along with the ncRNA ZNRD1-AS1, have been associated with disease states that are related to autoimmunity, immunity, and infection; while EPM2A and SHPRH have been associated with glycogen metabolism, cancer, and chemical dependency (Hindorff et al., 2013; Becker et al., 2013). In addition to this, a conserved regulatory element, NAT, was also found in these two blocks. NATs are an evolutionarily conserved group of ncRNAs that have been shown to mediate a number of cellular processes ranging from epigenetic modifications to regulation of transcription and post-transcription of protein coding genes in a multitude of species (Scherbakov and Garber, 2000; Havilio et al., 2005; David et al., 2006; Wang et al., 2005; Jen et al., 2005; Chen et al., 2004; Yelin et al., 2003; Zhang et al., 2006; Kiyosawa et al., 2003; Wang et al., 2008; Li et al., 2006, 2008; Sun et al., 2006; Chan et al., 2006; Rosok and Sioud, 2005; Imamura et al., 2004; Sleutels et al., 2000; Lee et al., 1999; Khochbin et al., 1992; Krystal et al., 1990; Munroe and Lazar, 1991; Enerly et al., 2005; Mihola et al., 2007; Volk et al., 1989; Kiyosawa et al., 2005; Kumar and Carmichael, 1997; Rossignol et al., 2002; Lapidot and Pilpel, 2006). St. Laurent and Wahlestadt (2007) have proposed that throughout evolutionary history, ncRNAs have experienced dramatic expansions that were in concert with increased organismal complexity. Pang et al. (2006) have shown that between mouse and human the sequence homology of the NATs (less than 70%) is equivalent to the sequence homology present in introns. This relaxation of evolutionary constraint may allow NATs to evolve at a faster rate as compared to other ncRNAs (Qu and Adelson, 2012). Studies suggest that the transition from unicellular organisms to multicellular organisms may have been possible due to the pervasive incorporation of ncRNAs into the genomes of early multicellular organisms (Gagen et al., 2005; Taft et al., 2007; Mattick, 2004, 2007). Georges St. Laurent et al. (2007) regard ncRNAs as “molecular information processors” that enhance the performance of cellular processes by integrating high density information between functional networks thereby faciletating the refined incorporation and collaborative action of many different molecular machines. For every species that has been sequenced to date, there is a correlation between organismal complexity and the number of ncRNAs (Taft et al., 2007), which has led to the view that ncRNAs are central to the information processing of complex organisms (Mattick, 2007; St. Laurent and Wahlestadt, 2007).

Moreover, we observed that Blocks 3013 (YRI) and 7016 (CEU) had genes that bind zinc ions and contain zinc finger (ZNF) domains. Zinc is a heavy metal that is an essential structural component of many proteins which include intracellular signaling enzymes and transcription factors (Vallee and Auld, 1993; Prasad, 1995). Zinc and ZNF domains play an important role in a number of biological functions, including wound healing, cellular communication, immune function, cell division, nucleic acid metabolism, cell replication, synaptic plasticity and protein synthesis (Classen et al., 2011; Sandstead, 1994; McCarthy et al., 1992; Solomons, 1998; Prasad, 1995; Fabris and Mocchegiani, 1995; Bitanihirwe and Cunningham, 2009; Murakami and Hirano, 2008). Zinc can be found in the brain, muscle, bone, kidney and liver (Wapnir, 1990; Pfeiffer and Braverman, 1982). The earliest use of zinc appears ~3.5 billion years ago (bya) and is believed to have been utilized as a messenger in nerve signaling, while the ZNF motif was associated with hormonal signaling (Williams, 2012). Likewise, it was noted that during times of rapid evolution, as seen in the Cambrian Explosion ~0.54 bya, dramatic expansion of ZNF domains occurred in response to the changing chemical composition of the sea shown by geochemical evidence of the accelerated rise of oxygen in the atmosphere, the increase in sulphate in the sea and the sedimentation of trace elements including zinc (Williams, 2012). This illustrates how life has adapted to its ever changing environment. In addition, the increase in the zinc content of proteins has been associated with the evolution of cellular complexity (Frausto da Silva and Williams, 2001; Williams and Frausto da Silva, 2006; Dupont et al., 2010; Zhang and Gladyshev, 2009).

In summary, the use of genomic energy units (GEUs) as a biophysical metric in SNP haploblock analysis has provided insights into the inherent structure and conservation of information in the human genome. We have demonstrated that highly favorable allelic potentials correlate with highly conserved genomic information units, in this case SNP haploblocks. Furthermore, the protein coding genes associated with the haploblocks of lowest block potential have strong homology across species underlining their fundamental role in the biological processes necessary for life. In addition to this, a conserved regulatory element and an evolutionarily conserved protein domain were also found in these blocks.

The development of genomic energy measures for the human genome relates the distribution of allele frequencies within a stable population to state variables associated with the environment within which that population resides. The state variables defined by the frequencies of common variants utilize the entropy of the statistical distribution of alleles to establish normalized information measures. Moreover, ‘genodynamics’ introduces more robust metrics for defining populations based on the genotypes of all individuals in the population as opposed to many current metrics based on the most frequent or common genotype in the population.

The NIC of the whole genome was found to determine an overall environmental potential that is a state variable which parameterizes the extent to which the environment drives variation and diversity within the population. Once this environmental potential (which is canonically conjugate to the entropy) has been determined, the genomic energies of individual alleles (nucleotides) and sets of alleles (haplotypes), as well as statistically averaged genomic energies for each persistent dynamic unit (haploblocks), can be directly calculated.

The assignment of genomic energies to alleles within a given environment allows the parameterization of specific environmental influences upon shared alleles across populations in varying environments. We are examining simple allelic dependencies on environmental parameters for future presentation.

## Figures and Tables

**Table 1:** Protein domains associated with functional genes located in block 3013 (YRI) and block 7016 (CEU). The protein sequences were scanned using PROSITE, a database of protein domains, families and functional sites. CDART, a domain architecture retrieval tool, was used to identify evolutionarily conserved domains which are in lowercase; while those in boldface are common in both blocks.

Protein domains	ZNRD1	PPP1R11	RNF39	EPM2A	FBXO30	SHPRH
carbohydrate binding module family 20 (CBM)				x		
Carbohydrate-binding-like fold				X		
Dual Specificity Phosphatase, Catalytic Domain				X		
protein-tyrosine/dual specificity phosphatase				x		
c-terminal helicase						x
Helicase, superfamily ½, ATP-binding domain						X
linker histone H1/H5, domain H15						x
P-loop containing nucleoside triphosphate hydrolase						X
SNF2-related Domain						X
WW Domain						X
**zinc finger**	x		x		x	x
f-box domain, cyclin-like					x	
TRAF-like domain					X	
b30.2/spry			x			
Butyrophilin-like			X			
Concanavalin A-like			X			
SPRY-associated			X			
SPla/RY anodine receptor SPRY			X			
protein phosphatase inhibitor		x				

**Table 2:** Molecular functions associated with the genes in block 3013 (YRI) and block 7016 (CEU). The molecular functions were determined by searching BioGPS, a gene annotation portal. Functions associated with one or more of the evolutionarily conserved protein domains are in lowercase, while those in boldface are common in both blocks.

Molecular functions	ZNRD1	PPP1R11	RNF39	EPM2A	FBXO30	SHPRH
Protein Binding				X		
protein ser/thr phosphatase activity				x		
protein try phosphatase activity				x		
protein ser/thr/tyr phosphatase activity				x		
starch binding				x		
ATP Binding						X
dna binding						x
helicase activity						x
ligase activity						x
**zinc ion binding**	x		x		x	x
ubiquitin-protein ligase activity					x	
DNA-directed RNA Polymerase Activity	X					
Nucleic Acid Binding	X					
protein phosphatase inhibitor activity		x				

**Table 3:** Biological processes associated with the genes in blocks 3013 (YRI) and 7016(CEU). The biological processes were determined using the web-based gene annotation tool, BioGPS. Those processes that are associated with one or more of the evolutionarily conserved protein domain are in lowercase.

Biological processes	ZNRD1	PPP1R11	RNF39	EPM2A	FBXO30	SHPRH
Behavior				X		
glycogen metabolic process				x		
Nervous System Development				X		
peptidyl-tyrosine dephosphorylation				x		
protein dephosphorylation				x		
DNA Repair						X
nucleosome assembly						x
protein ubiquitination						x
Nucleobase-Containing Compound Metabolic Process	X					
DNA-Dependent Transcription	X					

## Abbreviations

SNPs: Single nucleotide polymorphisms
LD: linkage disequilibrium
NIC: normalized information content
IC: information content
T_E_: environmental potential
GEUs: genomic energy units
YRI: Yoruba in Ibadan, Nigeria
CEU: Utah residents with ancestry from Northern and Western Europe
MHC: major histocompatibility complex
ZNRD1: zinc ribbon domain 1
ZNRD1-AS1: ZNRD1-antisense RNA1
HLA: human leukocyte antigen
HCG8: human leukocyte antigen complex group 8
PPP1R11: protein phosphatase 1 regulatory inhibitor subunit 11
HLA-J: human leukocyte antigen J
RNF39: ring finger protein 39
ncRNAs: non-coding RNAs
TFBS: transcription factor binding sites
miRNAs: microRNAs
NAT: natural antisense transcript
EPM2A: epilepsy, progressive myoclonus type 2, Laforin disease [laforin]
RP11-54515.3: uncharacterized protein
SNF2: sucrose nonfermentable 2
PHD: plant homeo domain
RING: really interesting new gene
E3: ubiquitin ligase
SHPRH: SNF2 histone linker PHD RING helicase E3 ubiquitin protein ligase
FBXO30: F-box protein 30
ZNF: zinc finger
bya: billion years ago
